# Rheumatoid arthritis and cardiovascular disease in relation to cytomegalovirus infection: a pilot cross-sectional study

**DOI:** 10.3389/fmed.2026.1926089

**Published:** 2026-08-27

**Authors:** Mohammed Ahmed Al-Kaif, Laith A. I. K. Al-Kaif, Zaid Saad Madhi, Dalya Talal Fathi Al-Azzawi, Wael Rasheed Obaead Alfatlawi, Sarah Kassab Shandaway Al-Zamali, Fatin Alaa Abdul Ameer Mahdi, Haibo Liu

**Affiliations:** 1 Department of Cardiology, QingPu Branch of Zhongshan Hospital Affiliated to Fudan University, Qingpu, Shanghai, China; 2 Department of Medical Microbiology, Hammurabi College of Medicine, University of Babylon, Hillah, Babylon, Iraq; 3 Department of Medical Rehabilitation and Physical Therapy, College of Health and Medical Techniques, Al-Mustaqbal University, Hillah, Babylon, Iraq; 4 Department of Family and Community Medicine, College of Medicine, Al-Iraqia University, Baghdad, Iraq; 5 Department of Medical Microbiology, College of Medicine, University of Babylon, Hillah, Babylon, Iraq; 6 Department of Surgery, Hammurabi College of Medicine, University of Babylon, Hillah, Babylon, Iraq

**Keywords:** biomarkers, cardiovascular disease, chronic inflammation, cytomegalovirus, rheumatoid arthritis, VEGF-A

## Abstract

**Background:**

Patients with rheumatoid arthritis (RA) face increased cardiovascular disease (CVD) risk not fully explained by traditional factors. Chronic cytomegalovirus (CMV) infection may contribute through immune activation and vascular inflammation. This pilot study investigated associations between CMV serostatus, vascular endothelial growth factor A (VEGF-A), and inflammatory markers in patients with RA and/or CVD.

**Methods:**

This cross-sectional study enrolled 76 participants (CVD = 40, RA = 18, RA+CVD = 18). Serum anti-CMV antibodies, VEGF-A, and IL-6 were measured by ELISA; CRP was assessed by agglutination; and CMV viral load was quantified by RT-PCR. Group comparisons analyses were performed.

**Results:**

CMV seroprevalence was 80.3% overall (CVD: 82.5%; RA: 77.8%; RA+CVD: 77.8%). VEGF-A levels were higher in CMV positive vs. CMV negative individuals, but the differences were not statistically significant; these data are inconclusive regarding a true association (e.g., overall mean: 67.8 vs. 44.5 pg/mL, *p* = 0.12). No significant differences were found among clinical groups for CMV IgG positivity, viral load, IL-6, CRP, or VEGF-A (*p* > 0.05 for all). However, viral load, IL 6 and CRP showed numerical increases that did not reach statistical significance.

**Conclusions:**

This pilot study is underpowered for definitive conclusions and should be interpreted as exploratory and hypothesis-generating. Although VEGF-A levels were higher in CMV positive individuals, the association was not statistically significant and the data are inconclusive. Larger, adequately powered studies are needed to clarify the role of CMV and angiogenic pathways in RA associated CVD.

## Introduction

Rheumatoid arthritis (RA) is a chronic autoimmune disease with substantial extra-articular manifestations, among which cardiovascular disease (CVD) is the leading cause of premature mortality, accounting for up to 50% of excess deaths (1, 2). RA patients have a 1.5- to 2-fold higher risk of myocardial infarction, stroke, and heart failure compared to the general population—a risk comparable to that of type 2 diabetes mellitus (3–5). Importantly, conventional risk factors do not fully explain this elevated risk (6); rather, persistent systemic inflammation, immune dysregulation, and endothelial dysfunction are key drivers of accelerated atherogenesis in RA (7). Pro-inflammatory cytokines such as TNF-α and IL-6 promote vascular damage and plaque instability (8, 9).

Chronic viral infections, particularly cytomegalovirus (CMV), have been implicated in perpetuating immune activation and vascular inflammation (10). CMV establishes lifelong latency and can periodically reactivate, expanding senescent T-cell subsets and sustaining cytokine production (11–13). CMV DNA and antigens have been found in atherosclerotic plaques, and CMV seropositivity has been associated with increased carotid intima-media thickness and adverse cardiovascular outcomes (14–16), though findings remain inconsistent (17).

Vascular endothelial growth factor A (VEGF-A), a key regulator of angiogenesis, plays roles in both RA synovitis and atherosclerotic plaque neovascularization (18, 19). Viral infections, including CMV, can induce VEGF expression via inflammatory and hypoxic pathways (20, 21), suggesting a possible mechanistic link between CMV and vascular pathology.

This pilot, exploratory study aimed to generate hypotheses regarding associations between CMV serostatus, VEGF-A levels, and the coexistence of RA and CVD in an Iraqi patient population. Given the exploratory nature of this work, we acknowledge that the study is underpowered for definitive conclusions and should be interpreted as hypothesis-generating.

## Materials and methods

### Study design and population

This investigation was designed as a pilot cross-sectional study to explore potential associations and generate hypotheses for future research. The study was conducted at Al-Imam Al-Sadiq Teaching Hospital in Babylon, Iraq, from September 2024 to March 2025. A total of 76 participants were consecutively enrolled and categorized into three groups: (1) CVD alone (*n* = 40), (2) RA alone (*n* = 18), and (3) RA+CVD (*n* = 18).

Inclusion criteria: age ≥18 years; diagnosis of RA per 2010 ACR/EULAR criteria; diagnosis of CVD (coronary, cerebrovascular, or peripheral arterial disease) confirmed by clinical assessment, imaging, or medical history; and written informed consent. Exclusion criteria: active malignancy, acute infections, immunosuppressive therapy (other than standard RA treatment), pregnancy/lactation, organ transplantation, or refusal to participate.

### Data collection

Demographic and clinical data (age, sex, disease duration, comorbidities, medications) were collected via interviews and medical records. Hypertension, diabetes, and hypercholesterolaemia were defined using standard clinical criteria.

### Laboratory measurements

Venous blood (5 mL) was centrifuged and serum stored at −80 °C until analysis.

- CMV serology: anti CMV IgM and IgG were measured using commercial ELISA kits (DiaPro, Italy). Primary CMV seropositivity was defined as detectable IgG. Reactive IgM results were noted but, given the well-documented limitations of IgM specificity in adult populations with chronic inflammatory conditions, were not used as the sole criterion for seropositivity. Confirmatory testing (e.g., IgG avidity) was not performed, which is acknowledged as a limitation.
- VEGF-A: quantified using a sandwich ELISA (R&D Systems) with a detection limit of 5.0 pg/mL; intra- and inter-assay CVs < 8% and < 10%.
- IL-6: measured by ELISA (Elabscience).
- CRP: detected by latex agglutination (Humatex CRP kit) with a cut-off of 6 mg/L.
- CMV viral load: DNA was extracted from 200 μL serum (Geneaid Viral Nucleic Acid Extraction Kit II) and quantified by real-time PCR (Bioneer kit) on an Exicycler™ thermal block. Standard curves ranged from 2 × 10^2^ to 2 × 10^6^ copies/mL.

### Statistical analysis

Analyses were performed using SPSS (SPSS Inc., Chicago, IL, USA) v26.0 and R v4.1.2. Continuous variables were expressed as mean ± SD or median (IQR) as appropriate; categorical data as frequencies (%). Group comparisons used univariate *t*-tests/Mann–Whitney *U* or ANOVA/Kruskal–Wallis with *post-hoc* corrections. A two-tailed *p* < 0.05 was considered statistically significant. Given the pilot nature and limited sample size, we did not perform multivariate regression analysis, as the number of covariates would exceed the recommended events-per-variable ratio. No adjustment for multiple comparisons was applied, and all *p*-values should be interpreted descriptively However, we have expanded our descriptive presentation of clinical characteristics to provide a more comprehensive overview of the study population.

### Sample size

The sample size was based on feasibility. With 61 CMV-positive and 15 CMV-negative participants, the study had 80% power to detect a large effect (Cohen's *d* = 0.65) at α = 0.05, but was underpowered for smaller effects. Furthermore, the number of statistical tests performed (comparisons across three groups and between CMV+ vs. CMV- individuals) increases the risk of Type I errors. No adjustment for multiple comparisons was made, and all *p*-values should be interpreted descriptively.

## Results

### Study population characteristics

Seventy-six participants (38 males, 38 females) were enrolled, mean age 52.4 ± 14.7 years. The majority were aged 30–60 years (59.2%) or >60 years (31.6%). Hypertension (36.8%), diabetes (28.9%), and hypercholesterolaemia (18.4%) were the most common comorbidities. Among RA patients (*n* = 18) and RA+CVD patients (*n* = 18), mean disease duration was 7.6 ± 2.8 and 12.7±4.3 years, respectively. Methotrexate was the most commonly used DMARD (94.4% in RA, and 44.4% in RA+CVD), followed by hydroxychloroquine (50.0%, 27.8%). Glucocorticoids were used by 44.4% of RA and 50.0% of RA+CVD patients. In the CVD and RA+CVD groups, Antiplatelet agents use was 45.0% and 83.3% respectively. In addition, statin use was 37.5% and 61.1%, respectively (Table 1).

**Table 1:** Demographic and clinical characteristics.

Variable	Total (*N* = 76)	RA (*n* = 18)	CVD (*n* = 40)	RA+CVD (*n* = 18)
Gender, *n* (%)				
Male	38 (50.0)	8 (44.4)	22 (55.0)	8 (44.4)
Female	38 (50.0)	10 (55.6)	18 (45.0)	10 (55.6)
Age, *n* (%)				
< 30 years	7 (9.2)	4 (22.2)	1 (2.5)	2 (11.1)
30–60 years	45 (59.2)	11 (61.1)	23 (57.5)	11 (61.1)
>60 years	24 (31.6)	3 (16.7)	16 (40.0)	5 (27.8)
Comorbidities, *n* (%)				
Hypertension	28 (36.8)	4 (22.2)	17 (42.5)	7 (38.9)
Diabetes mellitus	22 (28.9)	3 (16.7)	14 (35.0)	5 (27.8)
Hypercholesterolaemia	14 (18.4)	2 (11.1)	9 (22.5)	3 (16.7)
Smoking	7 (9.2)	1 (5.6)	5 (12.5)	1 (5.6)
RA disease duration (years), mean ± SD	10.2 ± 4.4	7.6 ± 2.8	–	12.7 ± 4.3
RA medications, *n* (%)				
Methotrexate	25 (32.9%)	17 (94.4)	–	8 (44.4)
Hydroxychloroquine	14 (18.4%)	9 (50.0)	–	5 (27.8)
Sulfasalazine	7 (9.2%)	4 (22.2)	–	3 (16.7)
Biologic DMARDs	8 (10.5%)	3 (16.7)	–	5 (27.8)
Glucocorticoids	17 (22.4%)	8 (44.4)	–	9 (50.0)
CVD medications, *n* (%)				
Statins	26 (34.2%)	–	15 (37.5)	11 (61.1)
Beta-blockers	23 (30.3%)	–	13 (32.5)	10 (55.6)
ACE inhibitors/ARBs	26 (34.2%)	–	15 (37.5)	11 (61.1)
Antiplatelet agents	33 (43.4%)	–	18 (45.0)	15 (83.3)

RA, rheumatoid arthritis; CVD, cardiovascular disease. –, N/A.

### CMV seroprevalence

Overall, 61/76 (80.3%) were CMV-seropositive (IgG+). Anti-CMV IgM was detected in 19.7%, and combined IgM/IgG in 22.4% (Table 2). Seropositivity was high across all groups: RA 77.8%, CVD 82.5%, RA+CVD 77.8% (Table 3), with no significant differences (*p* = 0.875).

**Table 2:** CMV serostatus distribution (primary definition: IgG+).

CMV serostatus	Total (*N* = 76)	RA (*n* = 18)	CVD (*n* = 40)	RA+CVD (*n* = 18)
IgG+ (primary definition), *n* (%)	61 (80.3)	14 (77.8)	33 (82.5)	14 (77.8)
IgG+ only, *n* (%)	44 (57.9)	10 (55.6)	23 (57.5)	11 (61.1)
IgM+ only, *n* (%)	0 (0.0)	0 (0.0)	0 (0.0)	0 (0.0)
IgM+/IgG+ (both), *n* (%)	17 (22.4)	4 (22.2)	10 (25.0)	3 (16.7)
IgM+ (any, supplementary), *n* (%)	17 (22.4)	4 (22.2)	10 (25.0)	3 (16.7)
Seronegative (IgG–/IgM–), *n* (%)	15 (19.7)	4 (22.2)	7 (17.5)	4 (22.2)

Primary CMV seropositivity is defined as IgG+ (61/76, 80.3%). IgM+ (any) includes both “IgM+ only” and “IgM+/IgG+” patients. In this cohort, no patient was IgM+ only; all IgM+ individuals were also IgG+. IgM results are presented as supplementary data and were not used as the sole criterion for seropositivity.

**Table 3:** CMV seropositivity across clinical groups.

Group	CMV+ Patients, n/N (%)
RA	14/18 (77.8)
CVD	33/40 (82.5)
RA+CVD	14/18 (77.8)
Total	61/76 (80.3)

CMV+ in this table refers to IgG seropositivity (IgG+), which is the primary definition of CMV infection used in this study. IgM results are presented separately in Table 2 and were not used as the sole criterion for seropositivity.

### VEGF-A levels by CMV status

**Figure 1:**
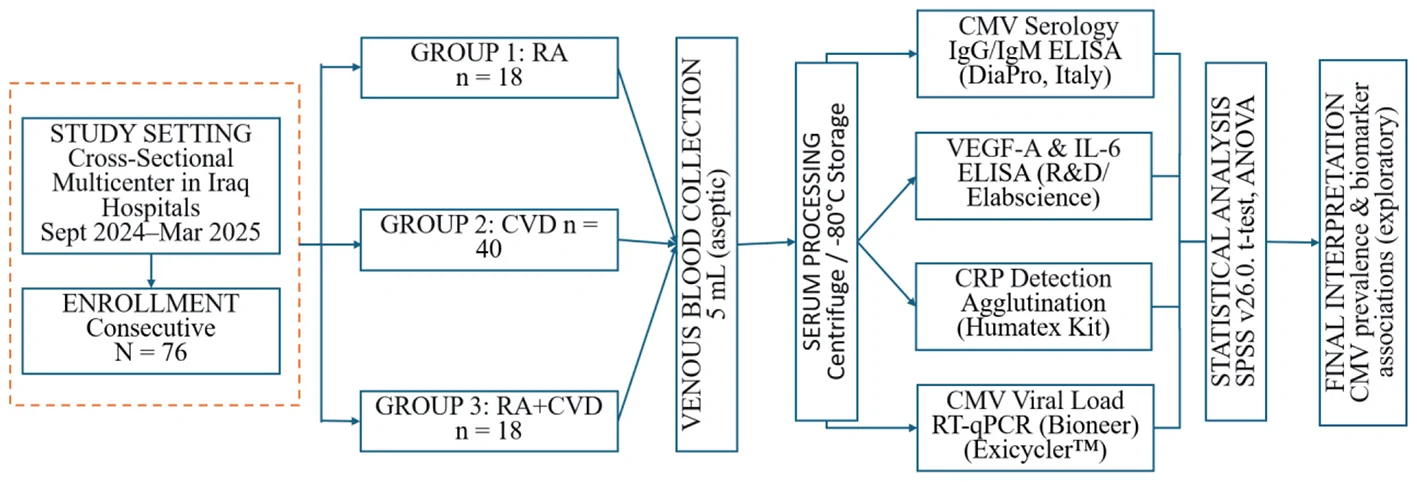
Flowchart of the study protocol. Schematic representation of participant recruitment, clinical grouping, biospecimen collection, laboratory measurements, and statistical evaluation.

Mean serum VEGF-A was higher in CMV positive compared to CMV negative individuals across all groups, but the differences were not statistically significant (overall: 67.8 ± 44.1 vs. 44.5 ± 28.9 pg/mL; mean difference 23.3 pg/mL, 95% CI: −2.7 to 49.3; *p* = 0.12). These findings are inconclusive and should be interpreted with caution. VEGF-A levels stratified by CMV status and clinical group are shown in Table 4 and Figure 1 illustrates the distribution of VEGF-A levels across study groups.

**Table 4:** VEGF-A levels stratified by CMV status and clinical group.

Group	CMV Status	*n*	VEGF-A (pg/mL), mean ±SD	*p*-value^*^
RA	Positive	14	71.8 ± 42.6	0.27
	Negative	4	43.1 ± 28.4	
CVD	Positive	33	72.9 ± 48.3	0.19
	Negative	7	48.9 ± 31.7	
RA+CVD	Positive	14	68.4 ± 39.2	0.31
	Negative	4	41.8 ± 26.5	
All groups	Positive	61	67.8 ± 44.1	0.12
	Negative	15	44.5 ± 28.9	

^*^Independent *t*-test comparing CMV-positive vs. CMV-negative within each group.

### Comparative analysis of biomarkers across clinical groups

CMV viral load, IL 6, and CRP levels showed numerical increases across the clinical groups, but these differences were not statistically significant (*p* = 0.063, 0.085, and 0.086, respectively). Given the limited sample size and multiple comparisons, these non-significant findings should not be interpreted as evidence of a biological trend. VEGF-A did not differ significantly between groups (*p* = 0.448; Table 5).

**Table 5:** CMV Serostatus, viral load, inflammatory biomarkers, and VEGF-A by clinical group.

Parameter	RA (*n* = 18)	CVD (*n* = 40)	RA+CVD (*n* = 18)	Total (*n* = 76)	*p*-value
CMV IgG+, *n*(%)	14 (77.8)	33 (82.5)	14 (77.8)	61 (80.3)	0.875
Viral load (copies/mL), mean ± SD	16,057 ± 47,189^#^	1,01,715 ± 1,57,966^##^	1,46,177 ± 1,75,039^###^	9,1531 ± 1,49,564^####^	0.063
IL**-**6 (pg/mL), mean ± SD	9.72 ± 6.85	17.30 ± 14.29	18.53 ± 15.28	15.80 ± 13.47	0.085
CRP (mg/L), mean ± SD	4.48 ± 3.88	8.94 ± 8.54	9.73 ± 9.06	8.07 ± 8.01	0.086
VEGF**-**A (pg/mL), mean ± SD	65.42 ± 35.47	68.72 ± 69.68	48.50 ± 36.69	63.15 ± 56.44	0.448

^#^*n* = 14.

^##^*n* = 34.

^*###*^*n* = 13.

^####^*n* = 61.

## Discussion

This pilot cross-sectional study investigated CMV seroprevalence and its association with VEGF-A and inflammatory markers in patients with RA, CVD, or both. As an exploratory investigation with limited sample size, our findings should be interpreted with appropriate caution and should not be considered definitive. Our principal findings are: (1) CMV seroprevalence is exceptionally high (80.3%) in this Iraqi cohort; (2) VEGF-A levels were numerically elevated in CMV-positive individuals across all disease groups, but this difference was not statistically significant; and (3) CMV viral load, IL-6, and CRP showed non-significant numerical increases with increasing clinical complexity.

The high CMV seroprevalence observed (80.3%) aligns with regional reports from Iraq and neighboring countries where CMV is acquired early in life (22, 23). Hussein et al. (24) reported rates of 85–90% among Iraqi populations. The comparable prevalence across RA, CVD, and combined groups may reflect the high background seroprevalence rather than a disease-specific association.

A methodological consideration is the use of IgG positivity as the primary definition of CMV seropositivity. While the inclusion of IgM in the definition did not substantially alter the overall prevalence (the same 61/76 patients were IgG+), we have maintained consistency by defining seropositivity based on IgG. IgM results are presented separately for completeness.

CMV infects endothelial cells, smooth muscle cells, and macrophages, promoting a pro-inflammatory and pro-thrombotic phenotype (25). Latent CMV infection is linked to expansion of senescent CD4^+^CD28^−^ T cells, which produce IFN-γ and perforin, contributing to endothelial damage (26). The comparable prevalence across RA, CVD, and combined groups may reflect the high background seroprevalence rather than a disease-specific association. In populations with near-universal CMV exposure, binary serostatus has limited discriminatory power, and more granular measures (e.g., antibody titres, T-cell responses) may be needed (27).

The observation of higher VEGF-A levels in CMV positive individuals, while not statistically significant, is consistent with a biologically plausible mechanism by which CMV could influence angiogenesis. However, the wide confidence intervals and small sample size mean that this finding is inconclusive. CMV immediate-early proteins can activate VEGF transcription via HIF-1 and Sp-1 (28), and CMV-induced inflammatory cytokines (TNF-α, IL-6) stimulate VEGF production (29). This mechanism could link chronic CMV infection to angiogenesis and vascular pathology. Previous studies have reported VEGF-A elevations in CMV-positive arthritis patients (30) and associations between CMV serostatus and VEGF polymorphisms in RA (31). The lack of statistical significance in our study likely reflects limited power due to the small sample, particularly the small number of CMV-negative participants (*n* = 15), rather than absence of a true effect.

The numerical increases in CMV viral load, IL 6, and CRP across clinical groups did not reach statistical significance, and these data should not be interpreted as evidence of a biological gradient. Viral load may better reflect ongoing viral activity than seropositivity alone. Low-grade CMV reactivation has been associated with chronic immune activation and vascular inflammation (32, 33). IL-6 is a major pro-inflammatory cytokine involved in endothelial dysfunction and atherosclerosis (34); CRP reflects systemic inflammation and cardiovascular risk (35). The parallel observed in our study support the hypothesis that CMV-related inflammatory activity may contribute to vascular pathology in RA, but this requires confirmation in larger cohorts.

Strengths include comprehensive serological assessment (IgG and IgM), quantitative VEGF-A and IL-6 measurements, and inclusion of three distinct clinical groups. The study also contributes data from an Iraqi population, where such information is scarce.

Our findings are consistent with studies showing high CMV prevalence in RA and CVD (36, 37), but the lack of significant association between CMV and VEGF-A echoes the uncertainty in the literature. Meune et al. (38) found that the independent contribution of CMV to cardiovascular risk in RA remains unclear. In high-seroprevalence populations, the signal from CMV-related pathology may be masked by background exposure, necessitating more refined immunological measures (39).

As a pilot study, these findings are hypothesis generating rather than confirmatory. Future research should employ larger, multicentre cohorts with adequate power, incorporate longitudinal follow-up, measure quantitative CMV antibody titres and CMV-specific T-cell responses, and adjust for comprehensive confounders. Including vascular imaging endpoints (carotid intima-media thickness, flow-mediated dilation) would help clarify mechanistic pathways. Ultimately, intervention studies (e.g., antiviral therapy) may be needed to establish causality.

Limitations are substantial. The lack of statistically significant findings in this pilot study should not be interpreted as evidence of absence of an association. The wide confidence intervals and small sample size (particularly the *n* = 15 CMV-negative participants) mean that meaningful differences may have been undetectable.

The cross-sectional design of this study is a fundamental limitation that precludes any causal inference. While we observed numerical differences in biomarker levels between CMV-positive and CMV-negative individuals, we cannot determine whether CMV infection precedes or contributes to these differences, or whether the observed associations are due to confounding factors. Reverse causality is also possible: patients with more severe RA or CVD may have immune dysregulation that predisposes to CMV reactivation. Similarly, we cannot determine whether CMV infection contributes to accelerated atherogenesis in RA or whether RA-related inflammation facilitates CMV reactivation. Prospective longitudinal studies are essential to establish temporal relationships and potential causality.

We lacked data on several potentially important confounders including RA disease activity scores (DAS28), Rheumatoid factor (RF), o Anti-CCP (ACPA), and BMI. Future studies should incorporate these variables in multivariate models. CMV assessment was limited to binary serostatus; quantitative antibody titres or T-cell phenotyping would provide more insight. The single-center, tertiary-hospital setting may limit generalizability. Finally, we did not assess other herpesviruses that might contribute to cumulative viral burden.

A methodological limitation is our inclusion of IgM results in the definition of seropositivity. In adult populations with chronic inflammatory conditions such as RA and CVD, false-positive IgM results are well-documented and can occur due to rheumatoid factor, polyclonal B-cell activation, or cross-reacting antibodies (40, 41). Without confirmatory testing (e.g., IgG avidity or immunoblot), we cannot distinguish true recent infection/reactivation from nonspecific IgM reactivity. However, when IgG+ was used as the primary definition of seropositivity, the overall prevalence remained 80.3%, suggesting that the inclusion of IgM did not substantially alter the findings.

## Conclusions

This pilot study found a high prevalence (80.3%) of CMV infection among Iraqi patients with RA and/or CVD. Although VEGF-A levels were higher in CMV positive individuals, this finding was not statistically significant and the data are inconclusive. No significant differences were observed among clinical groups for any measured biomarker. These exploratory findings should be interpreted as hypothesis generating. Adequately powered longitudinal studies are needed to determine whether CMV contributes to RA associated cardiovascular disease.

## Data Availability

The raw data supporting the conclusions of this article will be made available by the authors, without undue reservation.
